# Study on the Diffusion and Optimization of Sucrose in Gaido Seak Based on Finite Element Analysis and Hyperspectral Imaging Technology

**DOI:** 10.3390/foods13020249

**Published:** 2024-01-12

**Authors:** Wenlong Li, Yu Shi, Xiaowei Huang, Zhihua Li, Xinai Zhang, Xiaobo Zou, Xuetao Hu, Jiyong Shi

**Affiliations:** Agricultural Product Processing and Storage Lab, School of Food and Biological Engineering, Jiangsu University, Zhenjiang 212013, China; 18896656873@163.com (W.L.); 28810290556@163.com (Y.S.); huangxiaowei@ujs.edu.cn (X.H.); lizh@ujs.edu.cn (Z.L.); zhangxinai@ujs.edu.cn (X.Z.); zou_xiaobo@ujs.edu.cn (X.Z.); xuetaojsu@ujs.edu.cn (X.H.)

**Keywords:** finite element analysis (FEA), Gaidao, hyperspectral imaging technology (HSI), steak marinating

## Abstract

As a traditional Chinese dish cutting technology process, Gaidao artificially create cuts embedded in the food surface by cutting through it with knife, a process that currently plays an important role in the beef marinating process. And different Gaidao processes directly affect the beef marination flavour and marination efficiency. This study is the first to propose the use of Hyperspectral imaging technology (HSI) combined with finite element analysis to investigate the effect of Gaidao process on the quality of marinated beef. The study was carried out by collecting spectral information of beef marinated with different sucrose concentrations and combining various pre-processing methods and algorithms such as PLS, BiPLS, iPLS, and SiPLS to establish a quantitative model of sucrose concentration in beef, and finally optimizing parameters such as the length, position and number of Gaidao by Finite Element Analysis (FEA), which showed that when marinated with 1.0 mol/m³ sucrose solution, the concentration of sucrose in all tissues in the Gaidao steak reached 0.8 mol/m³ and above, which greatly improved the diffusion effect of the marinade. This work provides new ideas and methods to optimize the beef marinade Gaidao process, which has important practical value and research significance.

## 1. Introduction

Steak has been an important source of protein and fat for humans since ancient times. In order to enhance the texture of steak, enrich its flavour when eaten and preserve it efficiently, steak is often treated with marinating [1] methods, such as the sausages, ham [2], bacon, etc. In conjunction with existing meat production processes, marinating has become an essential means of flavour processing. The essence of marinating is the diffusion of the marinade within the food, for example, salt marinating [3] is often used to facilitate storage and increase the durability of the steak. Before post-processing, such as cooking, steaming, or frying, steak is also marinated in homemade sauces [4] to give it a more distinctive flavour and to satisfy the tastes of different people.

The time required for marinating is an important factor which largely determines the safety and flavour of the steak product. Depending on the size of the steak to be marinated, it can take between 3 h and 3 days if the conventional dry or wet marinating method is used. There are methods of accelerating the marinating process by applied physical fields such as ultrasound [5], pulsed electric fields [6] and vacuum tumbling [7], but these are not suitable for home-based marinating with small sample sizes and unique marinades. For this scenario, Gaidao is a traditional Chinese technique that greatly facilitates the rate of marination, essentially increasing the contact area between the food and the marinade by cutting into the surface of the food without destroying the overall structure of the food, thus serving to enrich the flavour and beautify the food. In modern times, Gaidao have been widely used in food preparation and processing due to its advantage of artificially creating contact surfaces. In conventional food marination, due to the differences in physical parameters and tissue structure, the overall marinade concentration distribution of the food becomes more and more uneven as the marination time increases. However, in the Gaidao food marination process, the new cutting surface can be regarded as the marinade inflow surface to optimise the area with small diffusion coefficient and poor marination effect, so that the whole food can be marinated uniformly in a shorter period of time in fact, to improve the efficiency of food processing, and to save the time and the cost of labour. However, the effect of the Gaidao parameters (position, depth, width, and length) on marinade diffusion cannot be precisely quantified, so there is an urgent need to investigate a method that can assess the quantitative relationship between the Gaidao process and the dynamic diffusion of marinade. Finite Element Analysis (FEA) [8] is a method of simulating real physical systems using mathematical approximations. A simulation study of meat marinating dynamics is an effective means of predicting changes in marinade content and its spatial distribution.

When validating the simulation results, conventional physicochemical methods usually serve as a reference by detecting the average value of the marinade over the entire sample during the marinating process, which does not allow for the visualization of continuous changes in the marinade at different moments and sites within the steak. Hyperspectral Imaging (HIS) technology [9] combines traditional spectroscopic and imaging techniques to obtain spectral as well as spatial data for each pixel of the sample and convert the spectral information in the hyperspectral image into a visual signal, thus visually and effectively observing the dynamic process of the marinade during the Gaidao steak marinating process.

**Scheme 1:**
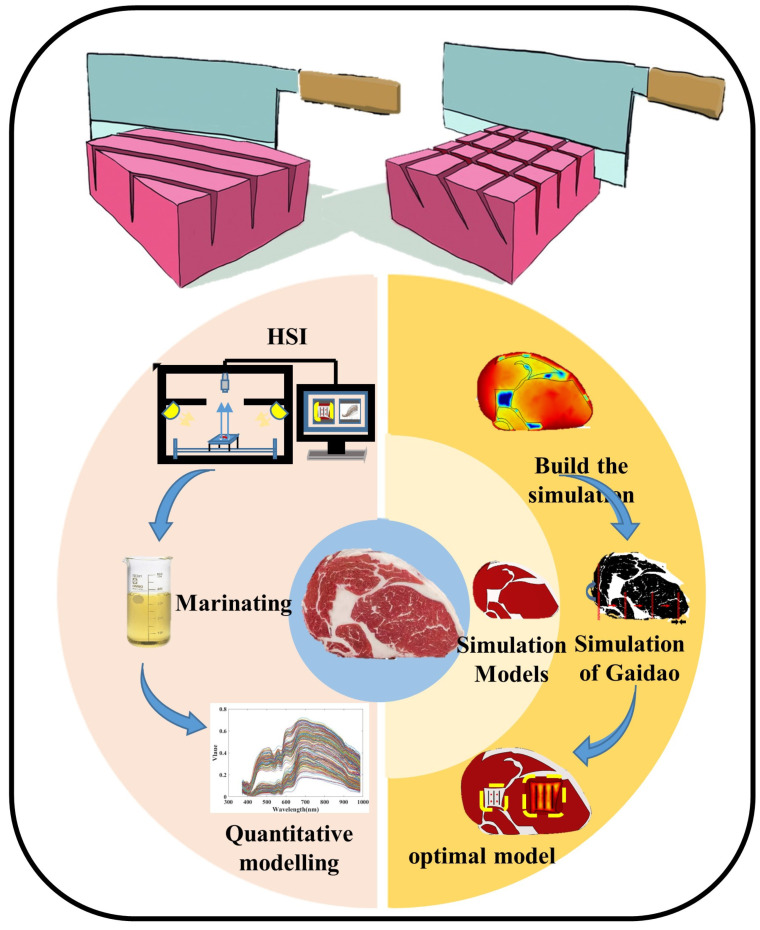
Schematic representation of the quantitative characterization of the marinating behaviour of Gaidao steaks.

To the best of our knowledge, this is the first report to use HSI combined with FEA to investigate the effect of the Gaidao process on beef marinating. In order to investigate the influence of various parameters during Gaidao and to provide a more intuitive reference for Gaidao foods rather than judging from experience. In this study, MATLAB binarized solid steak images were used to obtain modelling data, and using the resulting data to build a 3-dimensional model in COMSOL Multiphysics. Using COMSOL Multiphysics 5.6 with MATLAB to create materials, physical fields, etc., for marination by modifying various parameters within COMSOL by code within MATLAB. The optimal geometry of the Gaidao is then evaluated by changing the parameters in the actual marination and obtaining the concentration distribution in the steak after 36 h of marination. Finally, a quantitative marinade detection model was developed by using hyperspectral imaging to visualize the concentration of marinade in the steak, which was compared with the simulation results to verify the feasibility of the simulated Gaidao steak model and optimize its parameters to provide a reliable basis for the marination of Gaidao steak (Scheme 1).

## 2. Materials and Methods

### 2.1. Biological and Chemical Materials

Purchased a total of 100 fresh steaks with a clear separation of muscle and fat tissue and well-grained from Cameron’s Supermarket (Zhenjiang, Jiangsu Province, China) and the steaks was transported to the laboratory using ice pack-covered clamshells and stored in a −18 °C refrigerator under freezing conditions.

The sucrose purchased from Sinopharm Chemical Reagent Co., Ltd., (Shanghai, China) (C_12_H_22_O_11_, CAS: 57-50-1) and the sucrose solution is prepared from sucrose and plasma water.

### 2.2. Building a 3-Dimensional Model of Beef

MATLAB is an effective image processing software. The advantage of using MATLAB is that it can be combined with COMSOL software when performing finite element analysis, by writing code in MATLAB to control the parameters of the beef model, meanwhile the data obtained from simulations in COMSOL can also be iterated in MATLAB, greatly reducing the complexity of building models and data processing. Image binarization is a simple and efficient image processing method for clustering, which separates the target of interest from the background by setting the grey scale value of the pixels on the image to two grey levels of 0 or 255. Purchased beef consists mainly of muscle and fat tissue, and as the two tissues are distinctly different in colors, images of beef taken using binarization are helpful in building simulation models quickly.

### 2.3. Finite Element Analysis

#### 2.3.1. Determining the Physical Field and the Diffusion Equation

COMSOL Multiphysics [10] is a finite element multi-physics field numerical analysis software with arbitrary independent functions to control the solution parameters such as material properties [11], boundary conditions, loads and other parameters, as well as powerful mesh dissection capabilities. While phenomena simulated by the finite element method often originate in traditionally separate fields of applied physics and engineering, the method is also widely used in the food sector, mainly for drying and processing, storage and preservation of food products, simulation of temperature and humidity fields, etc. COMSOL has a complete internal MATLAB connection, which enables the control of the key parameters of the model to be programmed in MATLAB and the processing of the data from the simulation with complex algorithms. The beef marination process [12] is one of the chemical diffusions in the Dilute Matter Transfer module, i.e., the movement of a high concentration of marinade molecules to the interior of the beef at a low concentration. The diffusion of marinade during the marinating process can be modelled in this module using Equation (1).

(1) $$\frac{\partial y}{\partial x}+u\cdot \nabla c=\nabla \cdot \left(D\nabla c\right)+R$$

*D*: Effective diffusion coefficient of solute ($m^{2}/s$).

*R*: Reaction rate ($g/\left(m^{3}\cdot s\right)$).

*u*: Velocity vector (m/s)

#### 2.3.2. Materials for Simulation Models and Simulation Model Assumptions

In COMSOL Multiphysics, a simulated beef model is built with reference to the steak parameters obtained from image binarization, in which the material parameters of any tissue structure can be artificially set. To marinate the results of image binarization and reduce the complexity of the model calculation, the simulation model is set to a white fat region and a red muscle region. In this module, the transfer property equations for both muscle and fat tissues follow Equation (1). Shi Yu [13] has measured the transfer properties of muscle and fat in beef during his study of NaCl marination transfer in beef tissues, with the main factors being thermal conductivity, constant pressure heat capacity, density and diffusion coefficient. Referring to his research results, a diffusion coefficient of 2.4 × 10^−9^ ($m^{2}/s$) was set for muscle tissue and 7.9 × 10^−11^ ($m^{2}/s$) for fat tissue.

The following assumptions are made in the solution process:

(1) The shape of the model constructed by image binarization remains regular during the simulation
(2) The temperature is constant throughout the marinating process
(3) The concentration of marinade in all tissues of the whole beef at the start of the marination is 0
(4) Only the transfer of marinade between muscle and fat tissues is considered
(5) Ignoring the change in tissue structure and diffusion coefficient of the beef by marinade concentration
(6) Ignore the effect of physical fields other than dilute material transfer on marination

The calculation steps for the simulation process are as follows:

(1) Start COMSOL Multiphysics 5.6 with MATLAB software and invoke the Rare Matter Transfer module
(2) Import the steak model from MATLAB and optimize its edges
(3) Set material-related properties for the different tissue parts
(4) Divide the mesh and define the inflow surface of the marinade
(5) Set initial and boundary conditions for the model
(6) Set the solution step according to the simulated marinating time and solve for the transient process
(7) Analysis of simulation results and post-processing of results

#### 2.3.3. Optimization of Gaidao Parameters

The edges of the steak were identified by image binarization, the code for the cutting action on the meat in the Gaidao process was written in COMSOL Multiphysics 5.6 with MATLAB and the depression on the surface of the steak constructed by the cutting action was set as the new inflow surface for the marinade. The simulated marination time of the steak was 36 h. After calculation, the distribution of marinade concentration in the internal section of the simulated marinated beef model and the point concentration data of some 3-dimensional intercept points were derived as a reference, and the effect of this Gaidao model was evaluated by achieving 70% marinade concentration and above as a passing standard. Subsequently, code was written in MATLAB to continuously vary the depth, length and number of cutting surfaces, using the incremental and uniformity of the marinade as the evaluation criteria. The model that achieves the required marinade concentration and has the smallest cutting surface area is finally selected as the most optimal model.

### 2.4. Steak Marinated in Sucrose Solution

The usual material used for marinating is NaCl, which is made up of ions and its aqueous solution is an electrolyte solution, whereas sucrose is made up of molecules and its aqueous solution is a non-electrolyte solution. Hyperspectral imaging is sensitive to the content of organic components and if NaCl is used to marinate the steak, the concentration of the steak marinade can only be measured by conventional physicochemical methods. In order to highlight the differences in the diffusion of marinade molecules between the different components, this study used a 1 $mol/m^{3}$ sucrose solution to marinate beef and Gaidao beef. The sucrose marinated samples were divided into two parts, one for modelling the quantitative sucrose content within the steaks after marinating and the other for Gaidao according to the model obtained from the simulation and verifying the feasibility of the simulation by means of the quantitative sucrose model after the corresponding time of marinating.

Steak samples were processed for the construction of the sucrose quantification model: at an ambient temperature of 25 °C, 90 steaks were cut into 360 samples of equal size and uniform distribution of fat and muscle of 10.0 cm × 5.0 cm × 2.0 cm (Length × Width × Height) along the muscle fiber direction using a slicer. The steaks were defrosted to room temperature and then marinated for a total of 36 h. In groups of 5 samples, 30 min apart, the first group of samples was marinated for 30 min, the second group was marinated for 60 min and so on, and the spectral data were extracted to 72 groups of steak samples and the sucrose concentration was measured.

Samples treatment of Gaidao steak: Steaks with similar fat and muscle distribution to that in the optimal model obtained from the simulation were sliced longitudinally into blocks comparable to the model using a slicer, subsequently cut artificially on their surface to follow the shape of the optimal model, and finally marinated in a 1 mol/m³ sucrose solution for a definite time before being removed to extract the spectral data.

### 2.5. Hyperspectral Acquisition and Processing

#### 2.5.1. Hyperspectral Imaging Systems

In order to verify that the marinade of the Gaidao steaks is superior to that of the untreated steaks and to quantify the actual marinade concentration after the optimal Gaidao model, precise measurements of the marinade concentration inside the steak are required. Conventional chemical detecting methods usually detect the overall concentration within a sample up to a fixed point in the marinade and use this data as a reference value for the concentration at each location within the sample, without being able to effectively monitor changes in the concentration of the marinade in localized areas within the steaks. After the finite element analysis, more attention should be paid to the uniformity of the distribution of the marinade molecules inside the steak at the end of the marinade, with a low concentration of marinade, low increments of marinade and poor marinade being the target of the study. To overcome this problem, this study used HSI to detect the sucrose concentration in the steak after a certain time of marinating and to build a model for sucrose quantification, after which the spectral information of the Gaidao steak after marinating was extracted and brought into the model to verify the validity of the simulation results. HSI is an efficient and non-destructive detection method that can obtain complete spectral and spatial information of the sample and enable visual analysis of various components in the sample. The use of HSI allows for pixel-level monitoring of marinade concentrations [14], and comparison of this result with data obtained from simulations in COMSOL can indicate the feasibility of the Gaidao solution.

The hyperspectral images of the samples used in this study were all acquired in reflectance mode with a line scan HSI system in the wavelength range of 432 to 963 nm. The system at HSI is shown in Figure S1 and consists of a line scan spectrometer (ImSpector, VI0E, Spectra Imaging Ltd., Oulu, Finland), a CMOS camera (BCI4-U-M-20-LP, Vector International, Leuven, Belgium), two 150W illuminators (Fiber-Lite PL900-A, Dolan-Jenner Industries Inc., Boxborough, MA, USA), a transport drive system (Zolix TS200AB, Zolix.Crop, Beijing, China), a dark box (ZJgrt, Great Ltd., Beijing, China) and an industrial computer (HPdx2390MT, Hewlett-Packard, Beijing, China) with data acquisition and pre-processing software installed (Spectral Image, Isuzu-Optics Co., Hsinchu, Taiwan).

#### 2.5.2. Hyperspectral Data Acquisition and Extraction

Before acquiring the spectral data, the equipment was preheated 30 min in advance, the sucrose-marinated beef slices were laid flat on the conveyor table, the dark box door was closed, and the Spectral Image software was opened for hyperspectral image acquisition. Suitable conveyor belt speeds and camera exposure times were determined by several repeated experiments.

The acquired spectral image is a 3-dimensional block of data, which contains not only the spectral information of the sample, the image information but also a lot of background spectra. Therefore, a reasonable selection of a valid area for the study can reduce the amount of data processing operations and improve the accuracy of the quantitative model [15]. In this study, the rectangular tool in ENVI 5.2 was used to select the area around the center of the sample as the region of interest (ROI).

#### 2.5.3. Sucrose Concentration Detection

The average sucrose content in steak after sucrose marinating was determined by PAL-α Brix meter (ATAGO, Tokyo, Japan). Each sample was repeated three times and the mean value was used as data for the development of the quantitative model for sucrose.

#### 2.5.4. Visualization of Sucrose Content

One obvious advantage of hyperspectral imaging is the spatial imaging capability [16]. In order to provide a comprehensive and direct view of the differences in sucrose content within the Gaidao steaks, the best optimized model for each quality indicator was transferred to each pixel of the original ROI image. Calculate the dot product between the spectra of each pixel of the image and the regression coefficient of the best model and generate color maps where pixels exhibiting similar spectral characteristics at the information wavelength that have the same predicted value of the concentration index value [17]. The process was carried out in ENVI 5.2 and MATLAB 2016a.

## 3. Results and Discussion

### 3.1. Steak Model Marinade Simulation

**Figure 1:**
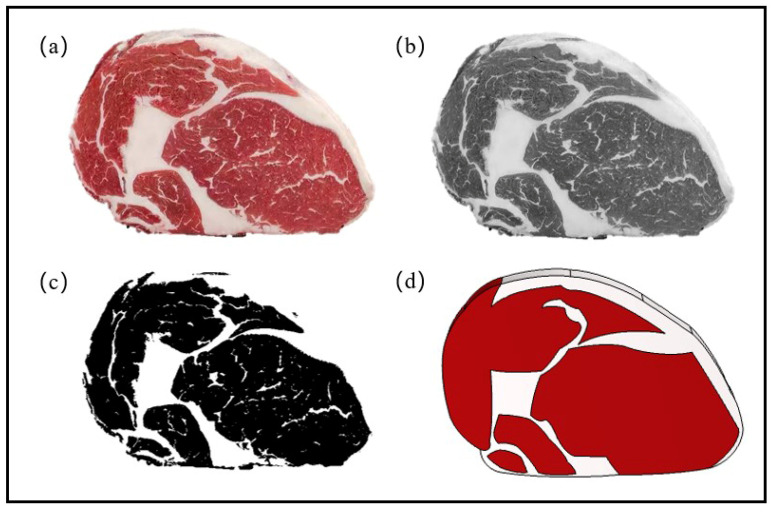
Binarized image and modelling results (**a**) Original RGB image (**b**) Grayscale image (**c**) Binarized image (**d**) COMSOL modelling results.

In order to obtain the specific parameters of the model to be simulated, the steak was divided along the cross-section into dimensions for cooking, measured by vernier calipers, as a 13.5 cm × 8 cm × 2 cm (Length × Width × Height) block of beef with flat top and bottom surfaces and regular edge lines. The segmented beef block was photographed in a light box and later the beef image was binarized by using MATLAB. As the tissue distribution of the segmented beef block was obvious and consisted mainly of two parts, fat and muscle tissue, the fascia and connective tissue were negligible in the steak, so only the hindering effect of fat and muscle on the transfer of sucrose molecules was considered in the COMSOL software simulation. In order to highlight the difference in marinade concentration between fat and muscle regions after a certain time of marination, the very small area of fat wrapped by muscle was ignored in the construction of the model, resulting in a schematic 3-dimensional model with a clear boundary between muscle and fat as shown in Figure 1d.

In the COMSOL finite element analysis, the movement of the marination belongs to the dilute matter transfer of the marinade molecules within the different components, and its movement rules follow Fick’s second law [18,19], which expresses the general relationship between the concentration of the diffusing elements and the time and position, according to the initial concentration of the elements and the corresponding boundary conditions, the specific concentration data of a position at the corresponding time point can be obtained [20]. According to Fick’s second law, the magnitude of the diffusion coefficient depends mainly on the temperature and pressure of the diffusing substance and the diffusion medium, and the diffusion coefficient [21], as one of the fundamental physical properties of a substance, represents the diffusion capacity of the material molecules. The diffusion coefficients of muscle and fat tissue in beef were set to be 2.4 × 10^−9^ ($m^{2}/s$) and 7.9 × 10^−11^ ($m^{2}/s$) [13]. 

**Figure 2:**
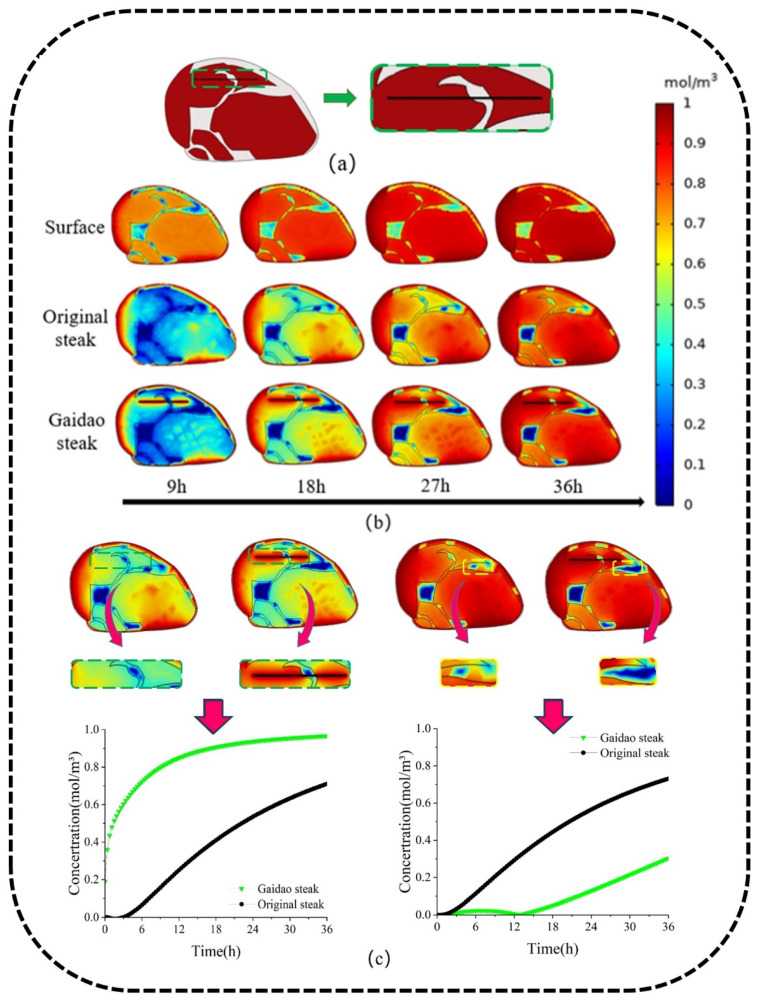
Marination effect of Gaidao and original steak model (**a**) Gaidao position (**b**) sucrose concentration distribution of Gaidao and original steak profiles over 36 h (**c**) Comparison of sucrose solution concentrations in selected areas.

In order to highlight the effect of Gaidao technology on steak marination, this study set the simulation time period from 0 to 36 h. Firstly, a model of Gaidao steak acting on both fat and muscle tissues was established as shown in Figure 2a, from which it can be clearly seen that new inflow surfaces were set within both fat and muscle tissues, while a model of original steak was used as the control group to observe the simulation of both models over a period of 36 h marination. An interval of 9 h was used to finally result in the distribution of sucrose molecules in the internal profiles of the models as shown in Figure 2b.

Comparing the two types of models at the beginning of the simulated marinade shows that the increase in the inflow surface within the steak due to the action of the Gaidao. The sucrose concentration near this inflow surface is higher than that at the corresponding location in the original model, but due to differences in the diffusion coefficients of fat and muscle tissue, the sucrose concentration in the fat tissue partially surrounded by muscle is much lower than in the surrounding muscle tissue. As shown in Figure 2c by counting the trends in sucrose concentration at the selected areas in the graph, it can be seen that at 18 h of simulated marinating, the Gaidao process had a significant effect on the diffusion of sucrose molecules in and around the area being acted upon., but sucrose concentrations at sites further away from this surface of the cut were not significantly different from the original model, instead there were a small number of muscle tissues in the Gaidao model where the concentration of marinade was less than in the original model, while at the same time the sucrose concentration in the fat tissue was already much lower than in the muscle tissue. By 27 h of marination, the sucrose concentration in the fatty areas within the original model was instead higher than in the Gaidao steak model, indicating that the Gaidao had a facilitating effect on localized areas of marination, but that the rest of the steak was simultaneously affected by irregularities and that an inappropriate Gaidao process could affect the overall uniformity of marination of the steak. By the later stages of marinating, with increasing marination time, sucrose molecules failed to diffuse well into the fat aggregation areas of the models in both models. It can be concluded that during the marinating process, the increment of sucrose molecules in the fat tissue is much lower than that in the muscle tissue because the diffusion coefficient of the fat tissue is lower than that of the muscle tissue, and the difference in concentration between the two tissues will gradually become larger as the marinating time increases; the diffusion coefficient of the fat tissue is the key factor affecting the uniformity of the marinating. The Gaidao process has a significant effect on the transfer of marinade molecules in the area being acted upon, but if the Gaidao process is carried out in an irregular and random manner, the uneven spread of marinade can occur, seriously interfering with the transfer behavior of the marinade in the steak as a whole and causing a great loss of flavor and quality to the marinated food. In response to these phenomena, parameters such as the number, position and length of actions in the Gaidao model are continuously improved and optimized, in the hope of obtaining a model with uniform marination of fat and muscle tissue and minimal Gaidao area.

### 3.2. Optimization of Gaidao Parameters

**Figure 3:**
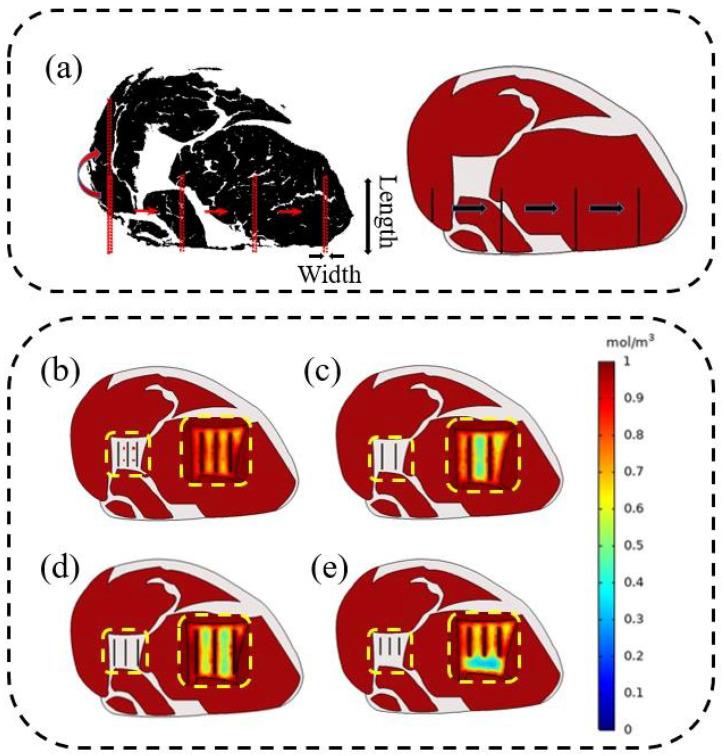
(**a**) Schematic diagram of MATLAB control COMSOL to optimize Gaidao (**b**) Optimal model for Gaidao (**c**) Control model for number of Gaidao (**d**) Control model for Gaidao position (**e**) Control model for Gaidao length.

For the above problem, the Gaidao is applied to the fatty areas of the steak that are more concentrated and surrounded by muscle. Meanwhile, in order to optimize the parameters of the Gaidao process, this study specifies that the most homogeneous marination model obtained with the smallest cutting surface area is the optimal model. Using COMSOL Multiphysics 5.6 with MATLAB, taking the binarization processed image as a template, when a white area in the image is identified (i.e., the area where the pixel point is identified as 1 in MATLAB), the action origin of the cut is set to be the coordinate point of the area, the cut width is fixed at 0.1 cm, the length initial value is 1 cm, the interval is 1 cm increments, the maximum value is the length of the image, and the number of reducers is incremented from 1 in that order. The model is simulated in COMSOL by writing a loop function in MATLAB as shown in Figure 3a, and the sucrose concentration distribution data of the profile at a height of 1 cm after each Gaidao simulation for 36 h. If there is an area of less than 70% sucrose concentration in the profile of this model, it is considered unqualified, and the next loop is executed. Until the concentration of marinade in the profile is above 70%, and the optimal Gaidao model with the smallest cut area is derived and waiting for validation. 

After simulation the optimal Gaidao model is shown in Figure 3b After simulation the optimal Gaidao model is shown in Figure 3b. In this model, a total of 3 cuts were made in the fat tissue, and the incision locations were evenly distributed in the fat aggregates. Three groups of Gaidao models with different parameters were also selected as control groups: a control model with the same Gaidao position and length and with twice cutting, a model with the same number of Gaidao but with larger intervals and a model with the same number and position of Gaidao but with different lengths. The four 3-dimensional cut-off points with significant variation in sucrose concentration were also selected on the same profile of the four groups of models to plot the variation in concentration from simulation 0 to 36 h to verify the marinating effect of the optimal Gaidao model.

**Figure 4:**
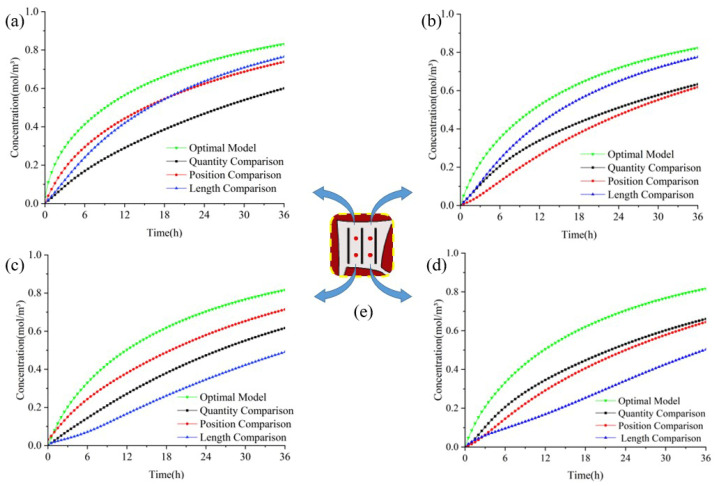
Plot of concentration change at 3-dimensional intercept points (**a**–**d**) Plot of concentration change at different 3-dimensional intercept points (**e**) Selection of 3-dimensional intercept points.

It can be concluded through Figure 4 that in four different models, when simulating a 1 mol/m³ sucrose solution marinating a whole steak marinated, Initially the steak is immersed in the sucrose solution and the concentration difference between the two parts is large, so the sucrose concentration increases faster at this time, while as the marinating progresses the concentration difference gradually decreases, the diffusive movement of the sucrose molecules also weakens, and a smaller increase in sucrose concentration can be observed in the image. The increase in sucrose concentration levelled off towards the end of the marinating period, when there is little difference between the internal and external sucrose concentrations in the steak. Comparing the 3-dimensional intercept point sucrose concentrations at different locations within the Gaidao area, it can be seen that for all four groups of models at any point in time at the same 3-dimensional intercept point, the sucrose concentration of the optimal model was greater than that of the remaining three control models, and the rate of increase in sucrose concentration was significantly higher than that of the other control groups. The sucrose concentration in any part of the optimal model exceeded 0.7 mol/m³ when the marinating time reached 36 h, thus proving the feasibility and rationality of the optimal Gaidao structure. The concentration distributions from the quantity-controlled and length-controlled models show that the contact area between the marinade and the steak is the main determinant of the marinating effect; the larger the contact area between the two sections, the faster the diffusion of the marinade molecules in the same amount of time. The concentration distribution plot of the position control model shows that a reasonably even Gaidao position is more conducive to the transfer movement of the marinade with the same contact area between the marinade and the steak, and that irregular Gaidao affects the uniformity of the marinade. In summary, COMSOL Multiphysics 5.6 with MATLAB can effectively simulate the marinating process of Gaidao steak, and by optimizing the Gaidao parameters we can obtain the optimal model with uniform marinating and smallest cutting area that we need. However, the actual marinating effect of the model needs to be further verified in conjunction with hyperspectral experiments.

### 3.3. Spectroscopic Data Analysis

Hyperspectral images contain hundreds of continuous bands of images, which contain not only the spectral information of the sample, but also a lot of useless background spectral information, making the spectral images large in data and redundant in information [14]. After acquiring the spectral information of a sample, different pre-processing methods are used to correct the full spectrum in time to eliminate scattering effects and useless high-frequency noise interference during the shooting process and to highlight the characteristic peaks in the spectral information [22]. Five different pre-processing methods were used in this study including standard normal variate information (SNVT), convolutional smoothing (Savitzky-golay, SG), multiplicative scatter correction (MSC), first derivative (1st-Der) and second derivative (2nd-Der) as shown in Figure S2. SNVT eliminates the effects of solid particle size, surface scattering and optical path variation on the spectra, mainly by centering and scaling the spectra, which results in smoother spectral profiles but makes some of the characteristic peaks harder to detect as shown in Figure S2d. The derivative pre-processing can provide higher resolution by smoothing out the interference from the background by eliminating baseline drift, overlapping peaks separately, and can be observed in Figure S2e,f as a more pronounced spectral change than the original spectrum, while the derivative calculation is often accompanied by an increase in noise that unnecessarily interferes with the spectral information [23]. Smoothing processing allows for the removal of random image noise with filters [24]. Multiple scatter correction processing is applied to remove non-linear baseline drift, thereby improving the stability and predictive power of the model.

### 3.4. Outlier Filtering

Monte Carlo (MOCD) is a statistical algorithm for detecting outliers by means of stochastic simulation, where the dataset is generally divided into a training set and a test set, and then a prediction model is built with the training samples according to a certain number of cycles to obtain the MV/STD plot of the prediction error. To ensure that all samples could be randomly selected multiple times, the number of cycles was set to 5000, with each cycle using the K-S algorithm to follow 80% of the samples drawn from the matrix as the training set and the rest as the prediction set. Finally, the deviation metrics MEAN and STD were calculated for each sample after prediction and scatter plots were drawn. The results are shown in Figure S3, where the majority of the scatter distribution is more concentrated, which represents the validity of the measured sample data. The outliers were split by dashed lines and a total of eight outlier samples were detected (12, 134, 135, 136, 151, 152, 162, 166). Removing the samples corresponding to the outlier markers for subsequent modelling analysis can effectively improve the robustness and predictive performance of the model.

### 3.5. Constructing a Quantitative Model for Sucrose

In this study, four different models for the quantification of sucrose in modified steaks after marinating were developed using each of the five pre-processing spectral data mentioned above and the raw spectral data. Statistics on the calibration and prediction sets of each model are shown in Table 1 reveal that the accuracy of the Backward interval Partial least squares (BiPLS) and Synergy interval Partial least squares (SiPLS) models is better than that of the Partial least squares (PLS) model, a multivariate calibration method that combines the advantages of principal component analysis, typical correlation analysis and multiple linear regression, and is currently the most used multivariate analysis method in quantitative analysis [25]. The results show that there is not only a certain correspondence between the sucrose content of the steak and the spectrum, but also many redundant signals and noise confounding, which affects the accuracy of the model to some extent. In contrast, Interval Partial least squares (iPLS) [26] does not take into account combinations between multiple spectral intervals, although it is more intuitive to filter the characteristic bands from the full band and find the band interval with the highest correlation to sucrose content [27]. SiPLS, on the other hand, is an improved interval selection algorithm based on iPLS, avoiding the loss of useful information and improving the correlation between spectra and sucrose content. In contrast, the feature variables screened by the BiPLS model corresponded best to sucrose content and the prediction model had the lowest RMSEP, which was superior to the other models in predicting sucrose content in steak. Combining the five pre-processing methods, the SNVT and MSC models for sucrose quantification performed poorly, in BiPLS the 2nd-Der pre-processing was the worst, even below the predictive performance of the original data, while the SG-processing model for sucrose content prediction in BiPLS performed best.

### 3.6. Visualization Analysis

**Figure 5:**
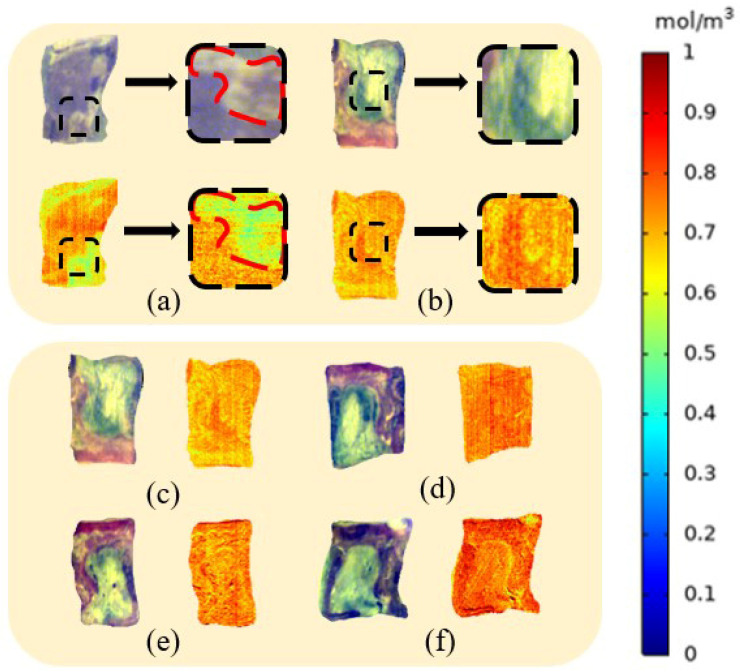
Visualization of sucrose content (**a**) Original beef visualization results (**b**–**f**) Gaidao beef visualization results.

The ROI regions of the Gaidao and original steak were first delineated by using ENVI5.2, after which the SG-BiPLS model was transferred to each pixel point of the ROI of the steak sample image using an imaging algorithm to generate a color map to vividly visualize the distribution of sucrose as shown in the Figure 5. In the original steak a noticeable difference in the concentration of fat and muscle tissue can be observed as shown in the dashed ticked area in Figure 5a, the internal sucrose distribution of the Gaidao steak was more uniform as shown in Figure 5b. This demonstrates the role of Gaidao in promoting and optimizing the diffusion of sucrose molecules during the marinating process, and provides a new idea for optimizing the marinating process and the Gaidao technique.

## 4. Conclusions

In this study, we propose to establish a 3D digital model of beef using image processing techniques and set up the model based on the physical property parameters of muscle and fat tissues, then PLS, iPLS, SiPLS and BiPLS quantitative detection models respectively for sucrose marinade in beef by HSI, in which SG-BiPLS has the best prediction effect with correlation coefficients of 0.94 for both the training and prediction sets. Subsequent parameter optimization of the Gaidao process using FEA combined with spectral image visualization demonstrated that the optimized model of Gaidao beef had a uniform distribution of sucrose and that the concentration of sucrose in both the muscle and fat fractions within the beef reached more than 80% after 36 h of marination. The method is applicable to the optimization of other Gaidao models and provides a reliable quantitative prediction and monitoring idea for Gaidao processes and multi-component meat marinating studies.

## Figures and Tables

**Table 1:** Quantitative modeling of sucrose content based on PLS, iPLS, SiPLS, BiPLS.

		Raw	1st-Der	2nd-Der	SG	SNVT	MSC
BiPLS	PLS factors	11	17	12	18	16	20
	Interval	{22 24 18 21}	{18 11 13 19}	{17 13 14 20}	{10 16 13 14}	{14 7 22 21}	{13 14 12 20}
	$R_{c}$	0.94	0.93	0.93	0.94	0.94	0.94
	RMSEC	2.51	2.6	2.72	2.56	2.51	2.50
	$R_{p}$	0.93	0.93	0.88	0.94	0.91	0.93
	RMSEP	2.68	2.75	3.52	2.72	3.06	2.73
iPLS	PLS factors	8	8	7	11	6	5
	Interval	{14}	{14}	{14}	{14}	{20}	{14}
	$R_{c}$	0.84	0.83	0.82	0.85	0.78	0.77
	RMSEC	4.09	4.08	4.21	3.96	4.61	4.7
	$R_{p}$	0.82	0.82	0.80	0.83	0.76	0.75
	RMSEP	4.33	4.31	4.51	4.15	4.82	4.99
PLS	PLS factors	{15}	{13}	{15}	{15}	{15}	{15}
	$R_{c}$	0.89	0.93	0.91	0.89	0.89	0.89
	RMSEC	3.28	2.73	3.00	3.29	3.41	3.44
	$R_{p}$	0.91	0.92	0.91	0.91	0.89	0.89
	RMSEP	3.17	2.83	3.10	3.22	3.33	3.41
SiPLS	PLS factors	11	15	11	10	13	13
	Interval	{14 17 18 20}	{13 14 16 19}	{13 14 17 20}	{14 17 18 20}	{8 13 17 20}	{8 13 17 20}
	$R_{c}$	0.94	0.94	0.93	0.93	0.94	0.94
	RMSEC	2.49	2.54	2.71	2.65	2.48	2.48
	$R_{p}$	0.93	0.91	0.88	0.92	0.90	0.89
	RMSEP	2.73	3.06	3.56	2.90	3.38	3.44

## Data Availability

Data is contained within the article.

## Supplementary Materials

The following supporting information can be downloaded at: https://www.mdpi.com/article/10.3390/foods13020249/s1.
