# Supplementary material for: Study on the Diffusion and Optimization of Sucrose in Gaido Seak Based on Finite Element Analysis and Hyperspectral Imaging Technology
Source: Foods. 2024 Jan 12;13(2):249. doi: 10.3390/foods13020249 (PMC10815083; doi:10.3390/foods13020249)
Supplement: Supplementary file 1 [file foods-13-00249-s001.zip › foods-2771794-supplementary.pdf]

# Supplement materials

# Study on the diffusion and optimization of sucrose in Gaido steak based on FEA and HSI

Wenlong Li, Yu Shi , Zhihua Li, Xiaowei Huang, Xinai Zhang, Xiaobo Zou, Xuetao Hu, Jiyong Shi*

Agricultural Product Processing and Storage Lab, School of Food and Biological Engineering, Jiangsu University, Zhenjiang, Jiangsu 212013, China

*Corresponding author:

Prof. Jiyong Shi

Tel: 139 1455 1781; E-mail: shi_jiyong@ujs.edu.cn

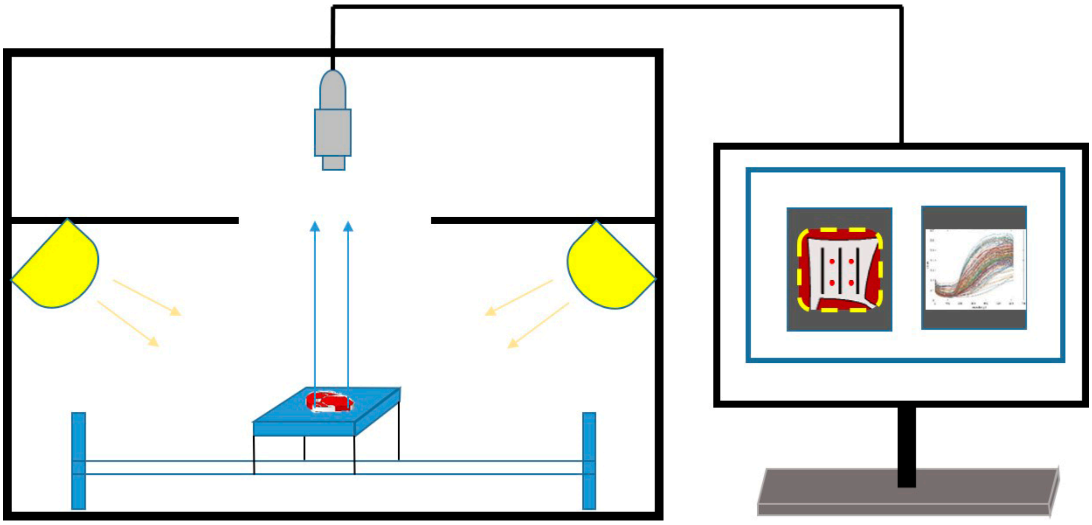

**Figure S1. Physical view of the hyperspectral image acquisition system**

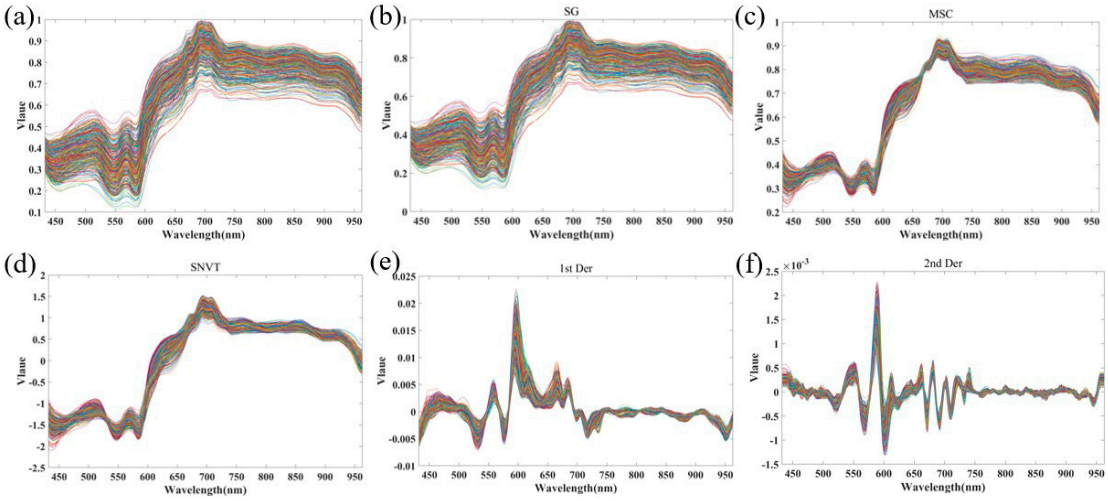


**Figure S2. (a) Raw spectra of sucrose marinated steak and (b) (c) (d) (e) (f) spectra after various pre-processing methods.**

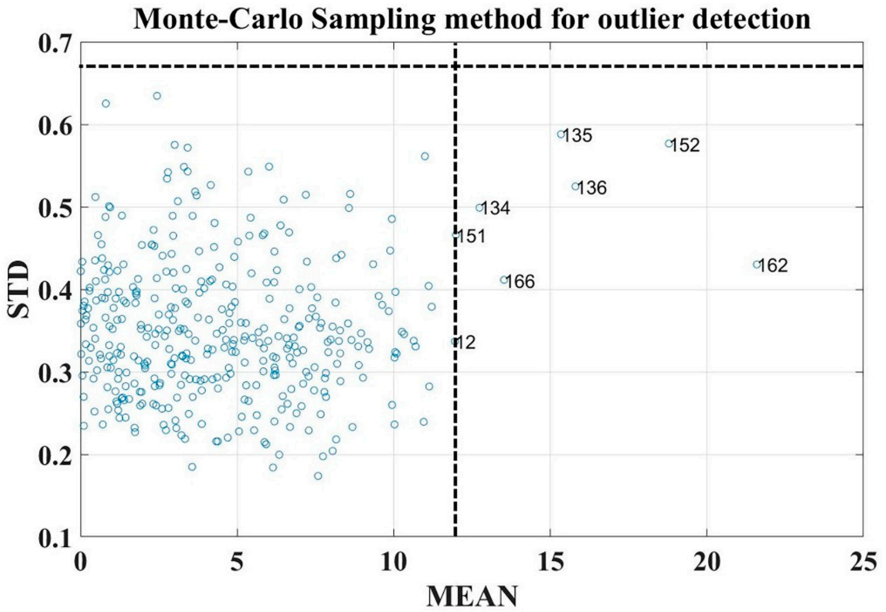


**Figure S3. Detection of outlier based on Monte Carlo method**
